# Automatic crack detection method for loaded coal in vibration failure process

**DOI:** 10.1371/journal.pone.0185750

**Published:** 2017-10-03

**Authors:** Chengwu Li, Dihao Ai

**Affiliations:** Faculty of Resources and Safety Engineering, China University of Mining and Technology, Beijing, China; MIT, UNITED STATES

## Abstract

In the coal mining process, the destabilization of loaded coal mass is a prerequisite for coal and rock dynamic disaster, and surface cracks of the coal and rock mass are important indicators, reflecting the current state of the coal body. The detection of surface cracks in the coal body plays an important role in coal mine safety monitoring. In this paper, a method for detecting the surface cracks of loaded coal by a vibration failure process is proposed based on the characteristics of the surface cracks of coal and support vector machine (SVM). A large number of cracked images are obtained by establishing a vibration-induced failure test system and industrial camera. Histogram equalization and a hysteresis threshold algorithm were used to reduce the noise and emphasize the crack; then, 600 images and regions, including cracks and non-cracks, were manually labelled. In the crack feature extraction stage, eight features of the cracks are extracted to distinguish cracks from other objects. Finally, a crack identification model with an accuracy over 95% was trained by inputting the labelled sample images into the SVM classifier. The experimental results show that the proposed algorithm has a higher accuracy than the conventional algorithm and can effectively identify cracks on the surface of the coal and rock mass automatically.

## 1. Introduction

Coal and rock dynamic disasters have become a great threat to the safe and efficient production of coal mines due to their sudden, rapid development, wide range and large degree of damage. Systematic research on coal and rock dynamic disasters has revealed that targeted monitoring means and preventive measures have already become a major problem in the field of coal mine safety.

The procedure of coal mining is bound to cause an internal stress response in the coal and rock mass, causing a local stress concentration or pressure relief and leading to instability and failure of the coal and rock mass. In the destabilization process, different stress states and stress levels will lead to different forms of coal and rock damage. The most direct manifestation of these failure modes is the production of cracks on the surface of the coal and rock mass. The accurate detection and analysis of these cracks can provide important guidance for preventing and controlling the destabilization of coal and rock and improving the safety of underground personnel. Accurate and timely detection of cracks in the front coal wall can effectively prevent coal-rock dynamic disaster in the production process of coal mines. Experienced workers often judge whether there is a possibility of coal and rock dynamic disasters through cracks on the coal wall. However, this method is often time-consuming and labour-intensive, and there is a certain degree of error and risk.

In recent years, with the development of electronic technology, an increasing number of image acquisition systems, such as industrial cameras, scanning electron microscopes, infrared thermal imaging technology and computer tomography technology, have been applied to coal and rock mass testing and field production as an effective monitoring means. At the same time, with the improvement of digital image processing technology, image segmentation algorithms based on threshold segmentation, edge detection and region growing are widely used in coal and rock image processing.

Cao et al. (2015) [1] proposed a modified C-V model based on the technique of image enhancement and obtained comprehensive crack information from coal and rock images. Crack images were obtained by electron microscopy and CT scanning and introduced fractal methods to analyse the cracks on the surface of the coal and rock mass (Li et al., 2014; Peng et al., 2015; Chen at al., 2010) [2–4]. Jiang et al. (2013) [5] studied the influence of primary cracks on the evolution of coal and rock shear failure. Xin et al. (2014) [6] studied the evolution law of coal deformation using digital image techniques.

At the same time, the application of digital image processing technology for surface crack and defect identification and analysis in many other areas for different objects (such as tunnels, food, bridges) has also received extensive research and development attention.

Cho et al. (2016) [7] investigated the effects of illumination and shooting distance on crack image recognition by examining cracks in images taken with a camera. Nashat et al. (2014) [8] proposed a pyramid automatic crack detection scheme. Zhang et al. (2014) [9] presented an automatic crack detection and classification methodology for subway tunnel safety monitoring. Abdel-Qader et al. (2016) [10] used the principal component principles (PCA) algorithm to extract cracks in concrete bridge decks for the purpose of automating inspection. Iyer et al. (2005) [11] presented a three-step method to identify and extract crack-like structures from pipe images whose contrast had been enhanced. Sinha et al. (2006) [12,13] developed a statistical filter for the detection of cracks in pipes and a simple, robust and efficient image segmentation algorithm for the automated analysis of scanned underground pipe images. Talab et al. (2016) [14] presents a new approach to image processing for detecting cracks in images of concrete structures. Li et al. (2014) [15] proposed a method consisting of three parts for bridge crack inspection. For the crack detection in a large structure, Sun et al. (2014) [16] presented a novel multi-scale algorithm for non-destructive detection of multiple flaws and (2016) [17] proposed a sweeping window method in elastodynamics for detection of multiple flaws embedded.

The above-mentioned methods have different properties for the detection of coal and non-coal surface cracks. The fractal and basic image segmentation methods can be used to mark the surface of the coal and rock mass accurately, but they cannot be used for rapid calculations. Many machine learning methods for non-coal surface cracks can be used to classify and analyse cracks effectively and accurately. However, it is worth noting that the special formation process of coal and later geological movement will result in a large number of primary cracks on the surface of coal and rock. Along with the coal roadway excavation, the surface of coal and rock will also partially shed and fold and presents complex features, such as discontinuity, non-uniformity, anisotropy and low contrast, which have a serious impact on the identification and marking of the surface of the coal and rock mass.

Therefore, considering the characteristics of surface cracks, a method of crack detection based on SVM is proposed for a coal and rock mass. A static load and static and dynamic combined loaded coal mass vibration failure test simulation system is built, and crack images of coal samples under the load conditions were obtained. Then, the images are sliced and labelled, and nearly 1000 samples are obtained. Then, 300 crack images and 300 non-crack images are selected as the experimental dataset. After the selection, 80% of the data set was randomly used for the training set, and 20% was used for the test set. Finally, the image is pre-processed and segmented; then, each segmented region is artificially identified and labelled "cracked" or "non-cracked", and 300 regions representing cracked and 300 regions representing non-cracked are obtained. After training through the SVM classifier and region data, a trained model is obtained, and the images are input to verify the algorithm.

## 2. Experimental

### 2.1. Experimental system

**Fig 1:**
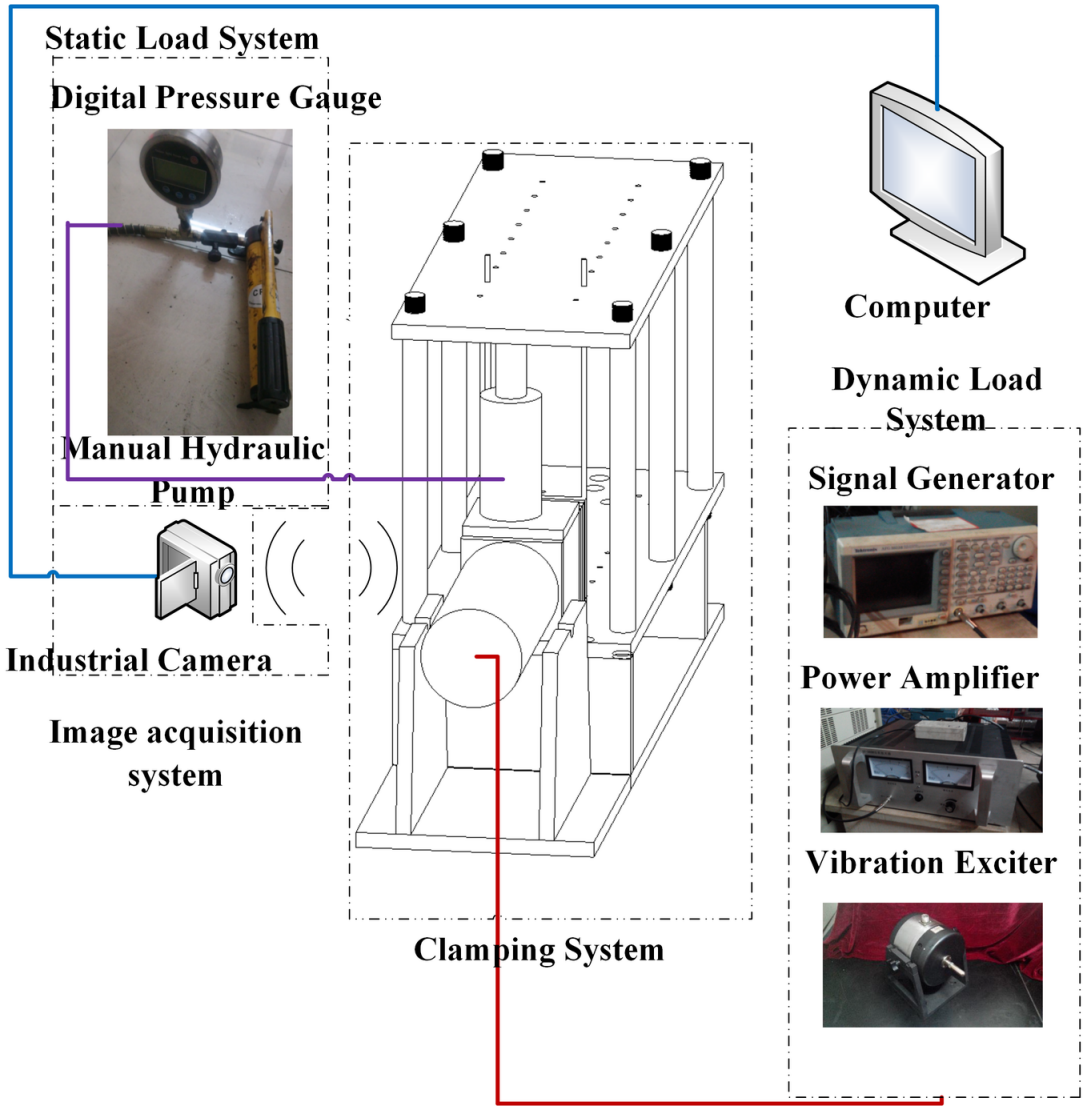
Schematic diagram of the experimental system.

**Fig 2:**
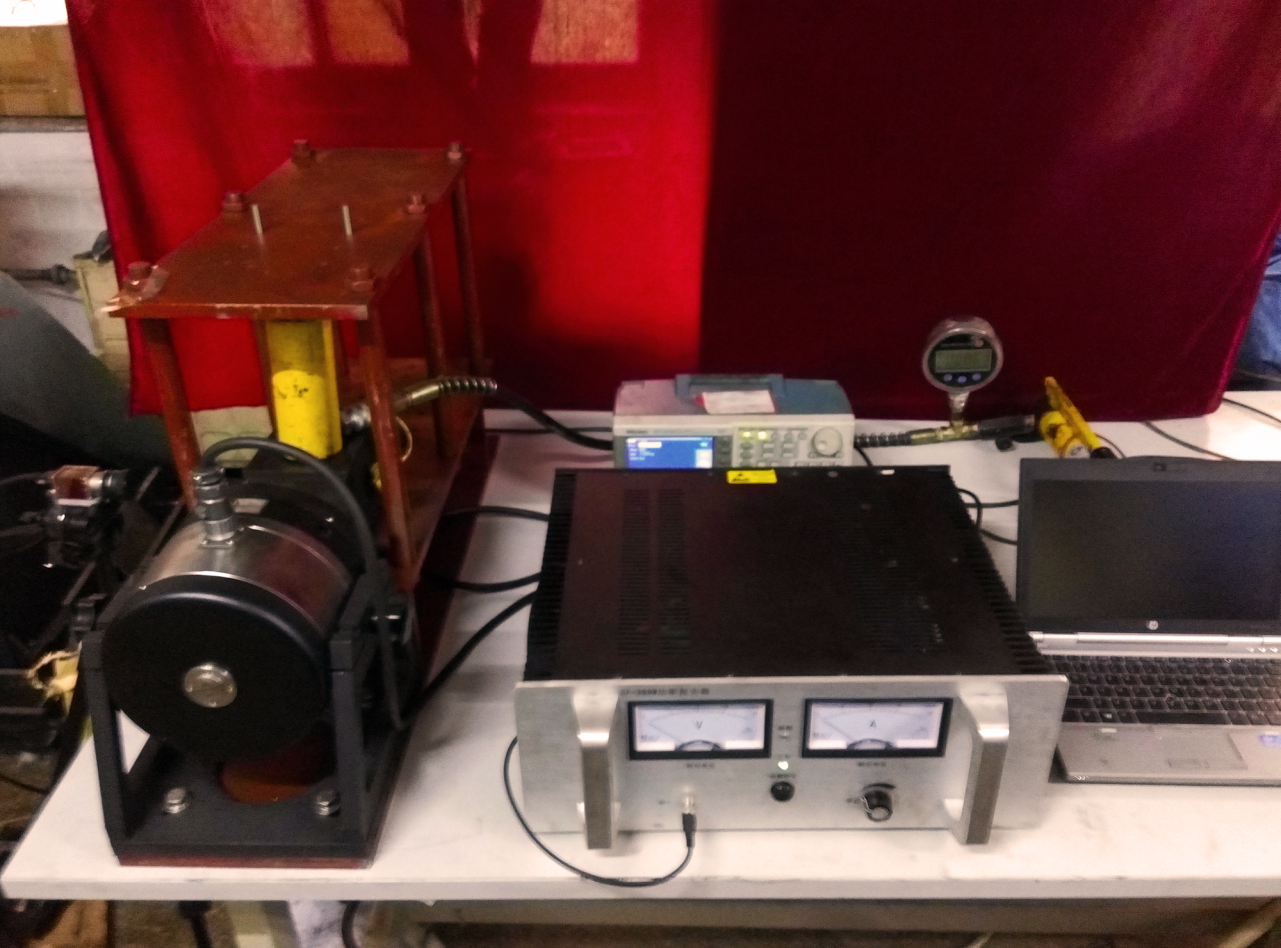
Physical diagram of the experimental system.

The vibration failure test system of the loaded coal is composed of a clamping system, a static load system, a dynamic load system and an image acquisition system. The role of the clamping system is to ensure that the coal specimen in the test will not offset while making the vibration wave propagation in the medium and the reflection effect on the boundary. To meet the test requirement, the clamping system is made of 201 austenitic stainless steel plates, and the acoustic impedance value is much greater than that of the coal. The static load system comprises a manual hydraulic pump, a jack and a precision digital pressure gauge. The purpose is to simulate the coupling stress field so that the coal can be in the critical state of failure. The dynamic load system comprises a signal generator, power amplifier, and a vibration exciter. The signal generator can generate sine wave, triangle wave, pulse wave, and square wave signals. The original signal from the power amplifier transmitted to the vibrator produces repeated vibrations. The image acquisition system uses the German Vision Technologies Allied company production of G-223B/C Mako industrial grade global exposure Gigabit Network Camera, data interface with IEEE 802.3 1000 baseT sensor types by CMOSIS CMV2000, camera resolution and maximum frame rate in the full resolution of 200 million pixels and 49.5FPS, can also be the edge trigger and edge triggered two ways. The schematic diagram and physical diagram of the vibration failure experimental system of loaded coal are illustrated in Figs 1 and 2.

### 2.2. Coal samples

**Fig 3:**
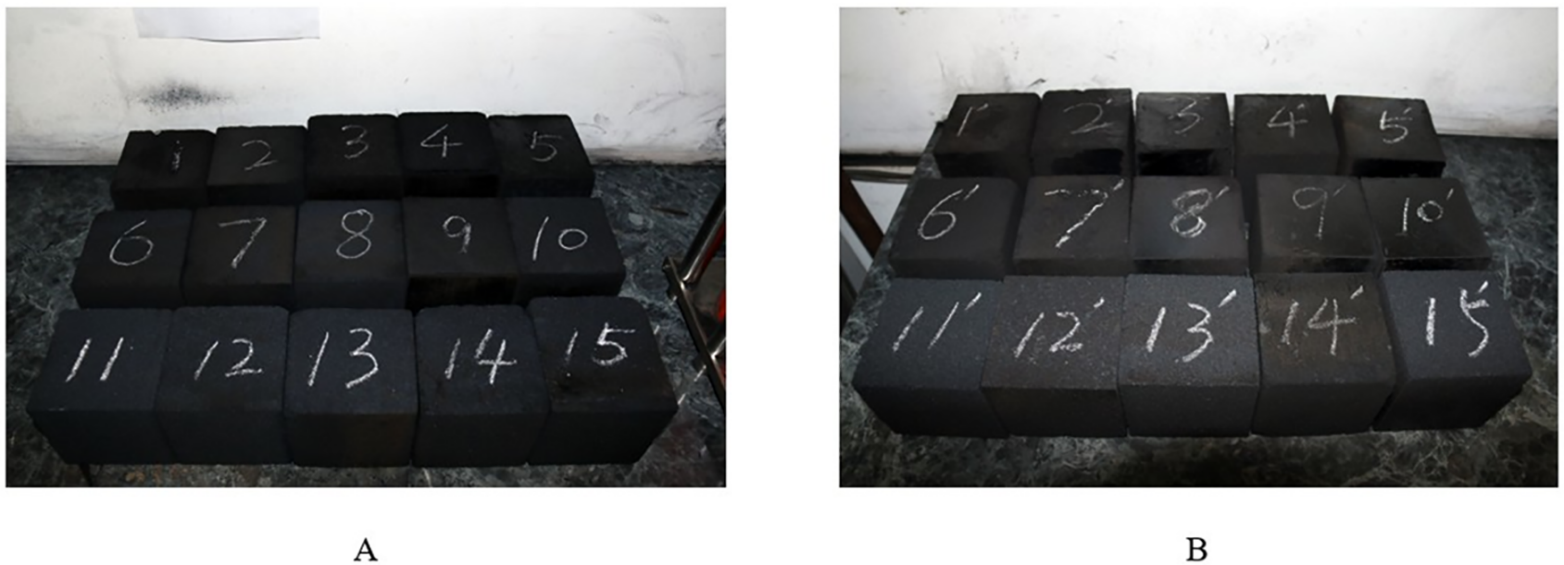
The physical map of briquette specimen.

To better simulate the occurrence of surface cracks in coal and rock under different conditions, two types of coal specimens are selected to represent soft coal and hard coal as shown in Fig 3.

The coal specimens used in this paper come from the TaShan Coal Mine Co., Ltd. and the PingDingShan Coal Mining Co., Ltd. (hereinafter referred to as the TS Coal Mine and PDS Coal Mine, respectively). In the reality, coal specimens with different particle sizes and hardness will produce different cracks. And the more crack types can improve the robustness of the detection algorithm. To this end, we made briquette specimens that has three different particle sizes: less than 0.25 mm, 0.25~0.5 mm, and more than 0.5~1.0 mm. The TS Coal Mine consistent coefficient is close to 1, belonging to solid coal, and the PDS Coal Mine consistent coefficient is far below 0.5, which indicates that the coal is soft.

**Fig 4:**
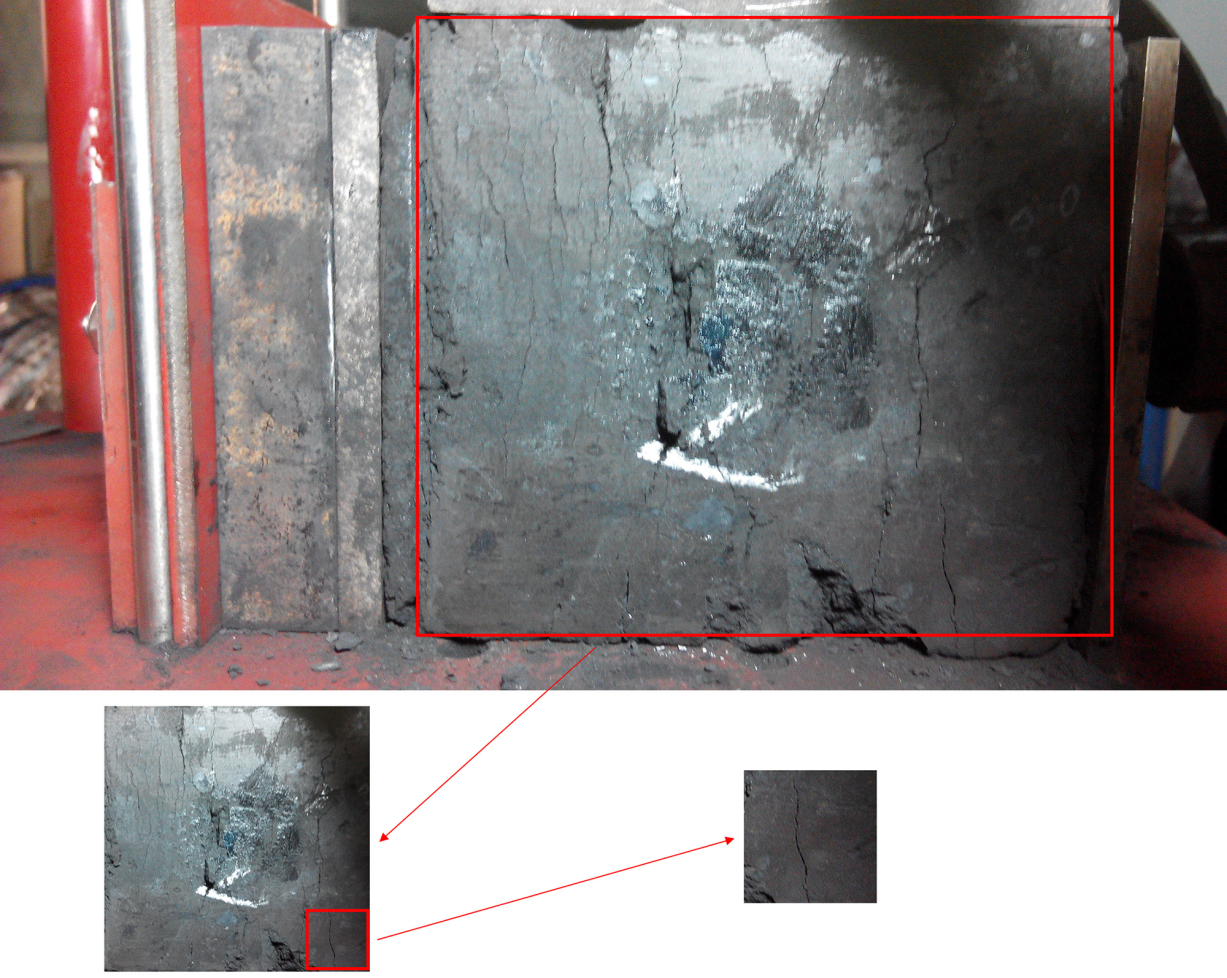
The selection process of the coal sample.

**Fig 5:**
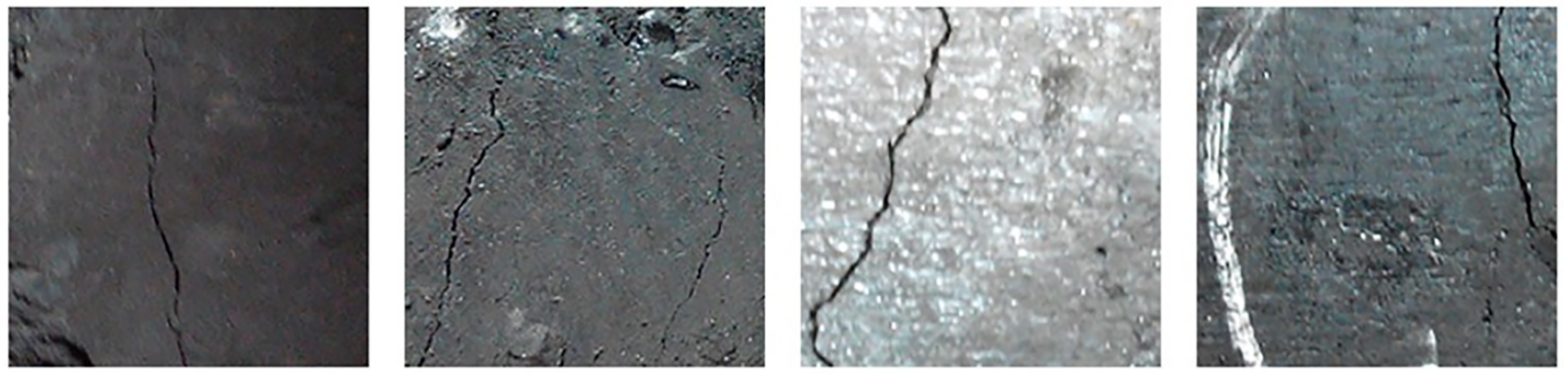
Examples of the surface of coal images with several types of cracks.

During the experiment, the surface of different coal bodies was recorded under different illuminations and angles, which could improve the robustness of the algorithm and the model. After the image is processed by region of interest (ROI), a 720 * 720 image of the surface of the coal body is obtained, and then, the surface image of the coal body is divided into 4 * 4 parts, where each pixel is 180 * 180. To illustrate the specificity of the surface of the coal and rock mass, the samples are selected as shown in Figs 4 and 5.

### 2.3. Experimental procedures

First, specimens are damaged by static load, which was recorded by pressure gauge reading synchronously; then, the specimen move into the critical state, and the explicit judgement basis will be discussed in another paper. At this time, there is much damage to the specimens, and the pressure gauge reading reaches the maximum, but there is no obvious crack. Maintaining the static load, open the vibration exciter to test at the same time, and transmit the image to the computer for analysis and storage.

To verify the accuracy and robustness of the algorithm, nearly 1,000 images were obtained in the experiment in this paper. Based on the characteristics of the crack described above, 300 cracked images and 300 non-cracked images were manually selected and labelled "1" and "-1", respectively. A training set will use 80% of the data, and a test set will use 20% of the data that was randomly selected.

Initially, the original image is directly input into the SVM classifier, and the recognition accuracy is not satisfactory or acceptable. After the threshold processing, the accuracy rate is only slightly increased. Therefore, a new method is proposed for crack identification.

After the image pre-processing and segmentation, the regions of each image are labelled from left-to-right and top-to-bottom according to the definition of 8-connected domains (Rafael C Gonzalez et al., 2013) (e.g., Region_1, Region_2, …, Region_n). Then, the eight features of each region are calculated. Then, 300 cracked regions and 300 non-cracked regions were selected manually as the data set and input into the SVM classifier.

**Fig 6:**
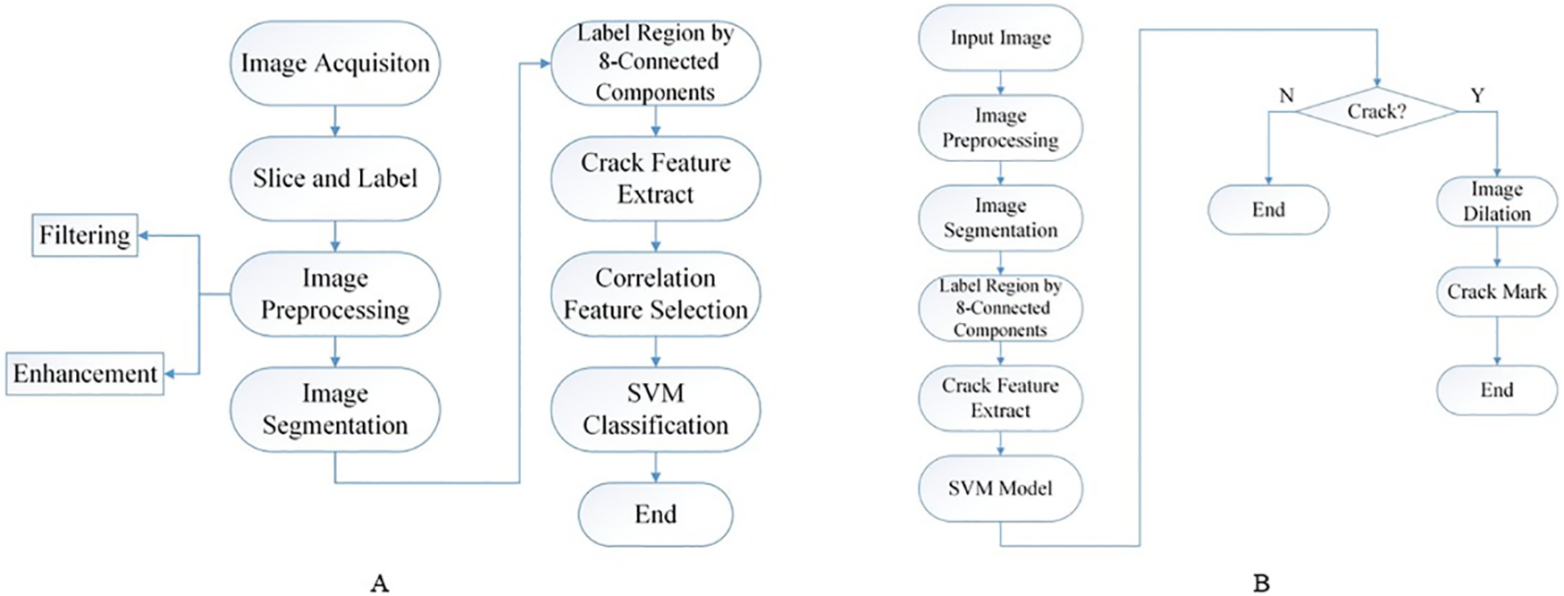
The experimental procedures. (A) The procedures of training a crack classification model. (B) The procedures of crack detection using the above model.

When we obtain a well-trained model, the crack image is processed by pre-processing and segmentation with the same parameters. The region number and the features of each region in each image are obtained and input into the SVM classifier for classification and prediction. Finally, the crack is marked and displayed by the method of dilatation. The Experimental procedure is shown in Fig 6.

For simplicity, the datasets of the image and region are denoted as:

$$\begin{array}{c} D_{image}=\left\{\left(x_{1},y_{1}\right),\left(x_{2},y_{2}\right),\ldots ,\left(x_{m},y_{m}\right)\right\},x_{i}=\left(x_{i1};x_{i2};\ldots ;x_{id}\right),\text{y}=\;\left(-1,\;1\right)\\ \text{m}=600,\;\text{d}=180\;*\;180 \end{array}$$

$$\begin{array}{c} D_{region}=\left\{\left(x_{1},y_{1}\right),\left(x_{2},y_{2}\right),\ldots ,\left(x_{m},y_{m}\right)\right\},x_{i}=\left(x_{i1};x_{i2};\ldots ;x_{id}\right),\text{y}=\;\left(-1,\;1\right)\\ \text{m}=600,\;\text{d}=8 \end{array}$$

## 3. Methodology

### 3.1. Image pre-processing

As shown in Fig 4, the surface of the coal body is not smooth; it is wrinkled and has a lower contrast. The image information is always subject to a certain degree of noise in the process of acquisition, quantification and transmission. The image quality is deteriorated, which will greatly affect the feature extraction and recognition. Therefore, it is necessary to improve the contrast between the crack and the coal surface and reduce the noise and other objects on the coal surface. The image filtering technique can be divided into the following two categories according to the space of processing: spatial domain method and frequency domain method [18].

**Fig 7:**
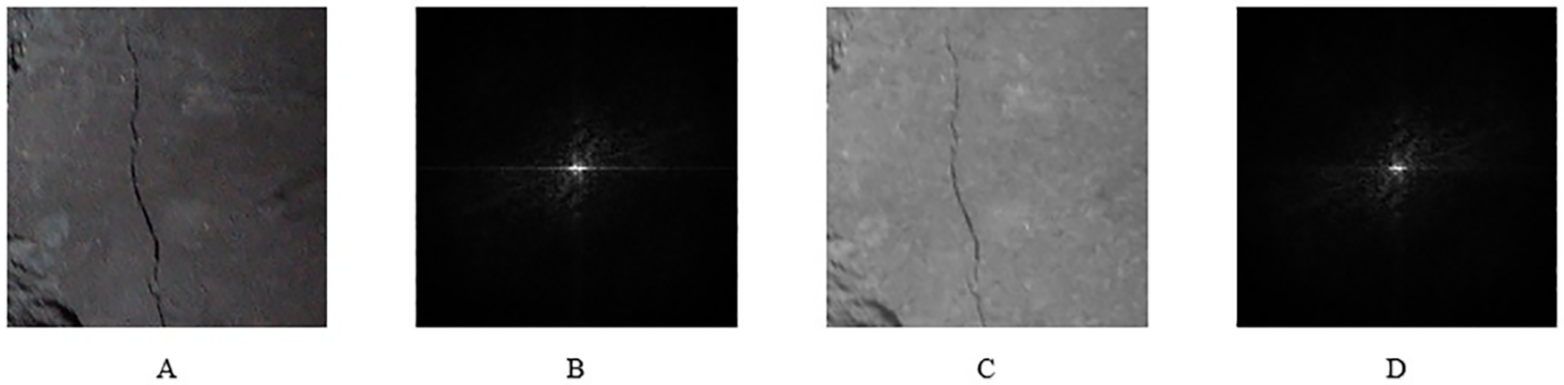
An example of a filtered image and its FFT. (A) The original image; (B) The FFT of the original image; (C) The image after a high pass filter; (D) The FFT of the image after a high pass filter.

As the crack on the surface of coal and rock is similar to the "edge" of the image, the frequency band belongs to the high frequency region of the image. Therefore, in contrast to several kinds of filtering, this paper chooses the high-pass filtering method. The filtered image and its FFT are shown in Fig 7.

After a high-pass filtering, low-frequency noise can be removed from some image surfaces. However, to better separate the image crack and background, further processing is required.

**Fig 8:**
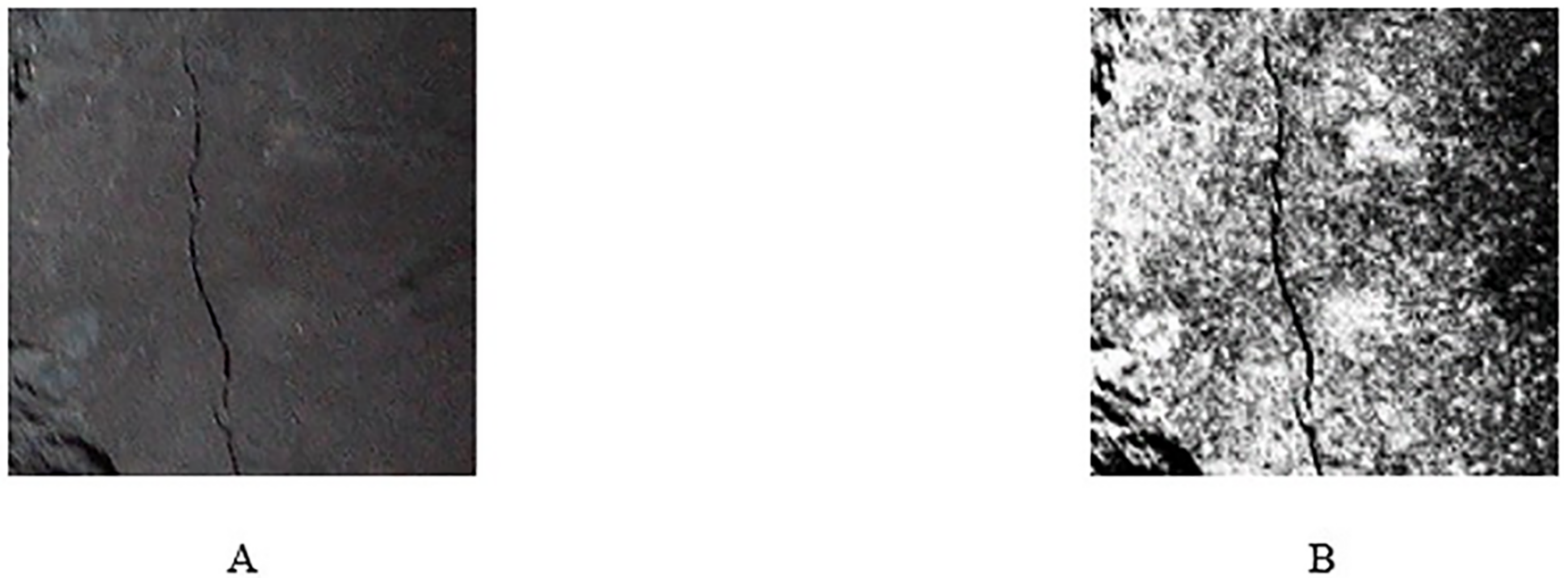
Image after histogram equalization processing. (A) The original image; (B) The image after the histogram equalization.

**Fig 9:**
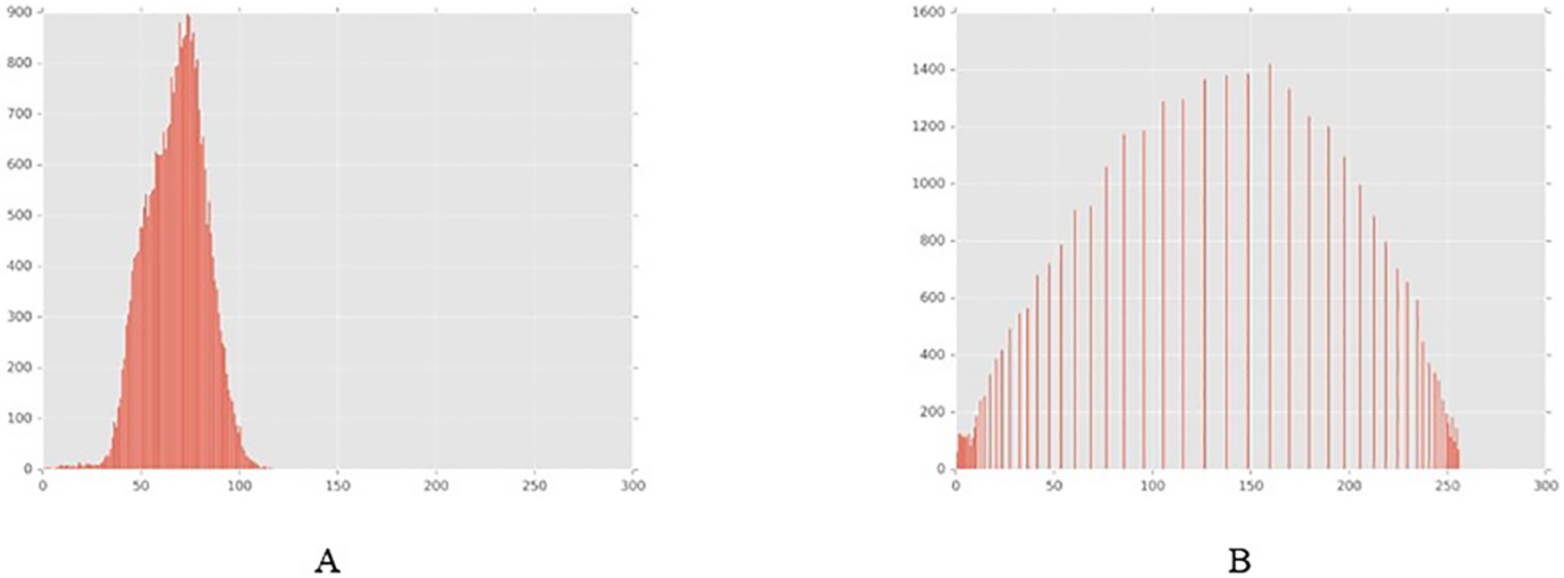
Image histogram. (A) The original image; (B) The image after the histogram equalization.

An image histogram allows us to understand the image profile, which will provide the basis for segmentation and threshold operations. Histogram equalization is a commonly used method where the central idea is to change the grey level histogram of the original image from a certain grey level to a uniform distribution in the whole grey scale. This method is often used to increase the overall contrast of an image, especially when the contrast of the useful data of the image is fairly similar. This method is useful for images that are too bright or dark in the background and foreground. The image after the histogram equalization processing is shown in Fig 8, and its histogram is shown in Fig 9.

### 3.2. Image segmentation

Image segmentation is a fundamental problem in image processing, and threshold-based segmentation is one of the most basic problems in image segmentation. Specific to the surface of coal and rock, because of the multi-texture, multi-target, weak signal property of the crack, and the variability of the image intensity and greyscale on the crack, it is difficult to obtain the optimal threshold by the threshold selection method. In threshold selection, a threshold that is too small will be extracted with other objects; in contrast, a threshold that is too large will miss the cracks in the image or make the crack become discontinuous.

Traditional image segmentation methods can be divided into the following three types: threshold method, region growing method and edge detection method [19]. The implementation principle of these methods is different, however they are based on low-level semantics of images such as the colour, texture, and shape of an image pixel. On the other hand, combining image intermediate and high-level semantics to enhance image segmentation effect has become a hot research topic in recent years. However, these intermediate and high-level image segmentation methods are suitable to complex images and situations, and they are time-consuming. And our goal is to segment crack from background. Therefore, in our work, we use the low-level semantics segmentation methods.

**Fig 10:**
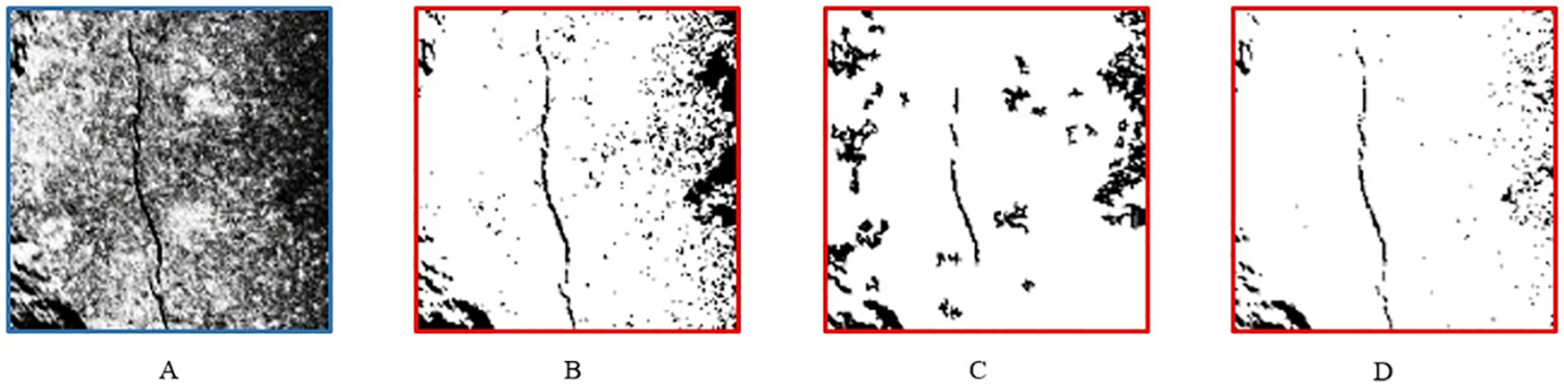
The result of other segmentation methods. (A) The original image. (B) The result of dual threshold. (C) The result of region growing threshold. (D) The result of hysteresis threshold.

As shown in the Fig 10, we choose three common segmentation methods from threshold method, region growing method and edge detection method: they are dual threshold, region growing threshold and hysteresis threshold.

**Fig 11:**
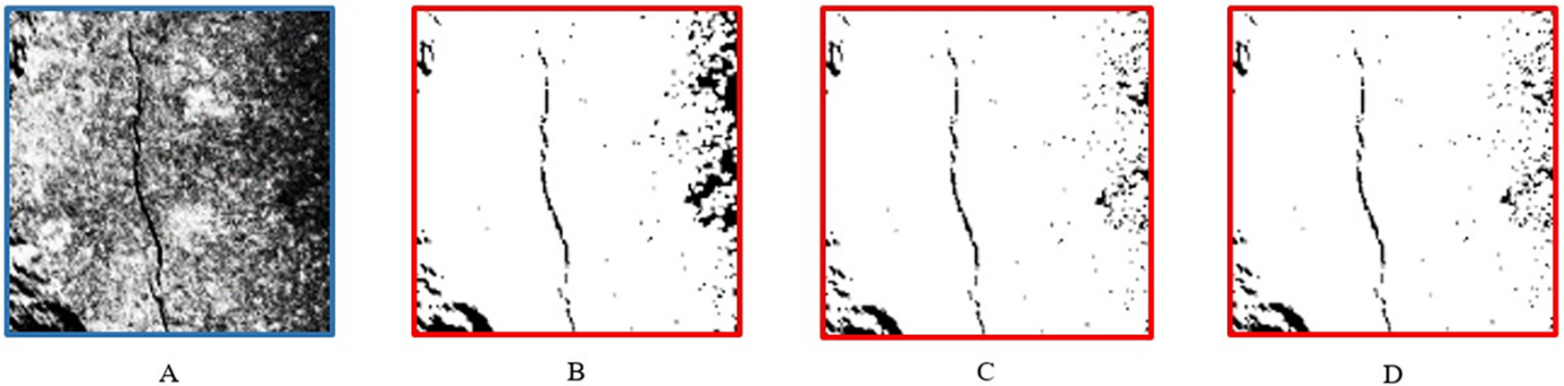
Examples of images after hysteresis threshold with different parameters. (A) The original image. (B) Min = 10, max = 60, length = 1. (C) Min = 10, max = 60, length = 5. (D) Min = 10, max = 60, length = 10.

The core of dual threshold is to segment an image into a region with grey values greater than a positive value and a region with grey values less than a negative value. And regions whose maximum grey value is less than minimal grey in absolute value are suppressed. The core of region growing is to segment an image into regions of the same intensity—rastered into rectangles. In order to decide whether two adjacent rectangles belong to the same region only the grey value of their centre points is used. Hysteresis threshold performs a hysteresis threshold operation (introduced by Canny J et al. (1986) [20]) on an image. All points in the input image having a grey value larger than or equal to a high value are immediately accepted (“secure” points). Conversely, all points with grey values less than low are immediately rejected. “Potential” points with grey values between both thresholds are accepted if they are connected to “secure” points by a path of “potential” points having a length of at most max length points. This means that “secure” points influence their surroundings. Therefore, we can adjust the maximum, minimum and length values to achieve the best segmentation results. Images with different parameters (low grey value, high grey value and max length are denoted by min, max and length, respectively) after hysteresis threshold processing are shown in Fig 11.

We can intuitively see that the Fig 11 has a better segmentation result than Fig 10 as it not only fully extracts the crack from the original image, but also removes most of the useless areas. Furthermore, we use the mean IU (Intersection over Union) [21] as a metric to quantitatively evaluate the effectiveness of different segmentation methods.

$$IU\left(R,\;R^{\prime }\right)=\;\frac{\left|R\cap R^{\prime }\right|}{\left|R\cup R^{\prime }\right|}$$ (1)

Where *R* denotes the segmentation results and *R*′ denotes the ground truth which means the region that segmented by the experts. The IU values of different segmentation methods are shown in Table 1.

**Table 1:** The IU value of different segmentation methods.

	|*R* ∩ *R′*|	|*R* ∪ *R′*|	IU(*R*, *R′*)
**Dual threshold**	1133	3638	0.31
**Region growing threshold**	855	3507	0.24
**Hysteresis threshold**	785	1133	0.69

### 3.3. Crack feature extract

As shown in Fig 11, after the image filtering, enhancement and segmentation, most of the unrelated regions have been removed; however, many unrelated regions are incorrectly retained; therefore, it is necessary to use the characteristics of the cracks to distinguish them.

From the previous definition of the crack, the surface crack of coal has the following characteristics: cracks were mainly horizontal or vertical strips; the crack length varies; a certain area may appear with multiple cracks; and as the static loading and dynamic loading pressure of the coal body increase, the crack width will increase.

**Fig 12:**
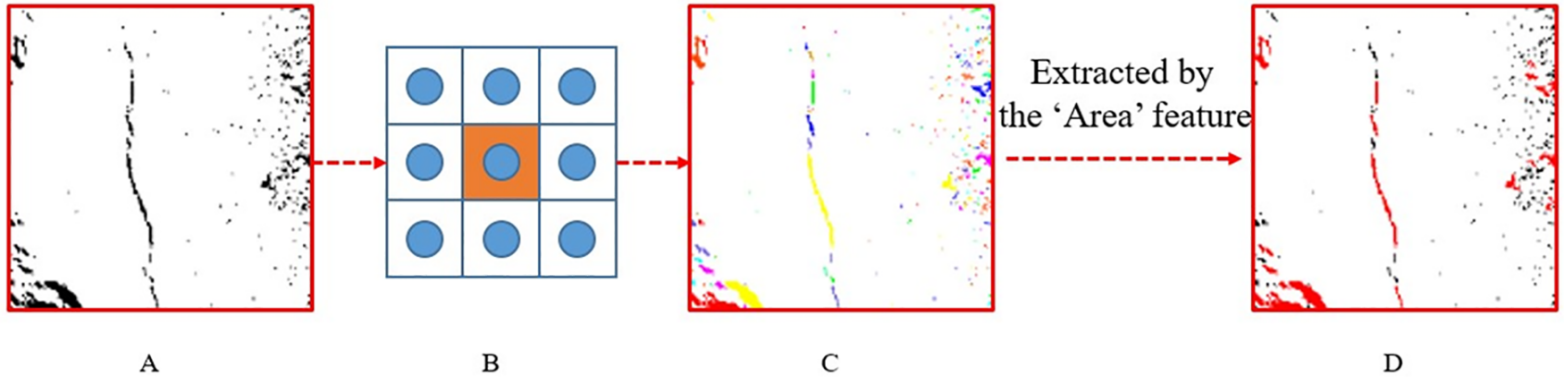
Procedure of crack ‘Area’ feature extracted. (A) The image after segmentation. (B) The diagram of 8-connected components. (C) The image after labelling based on 8-connected components. (D) The image extracted by the ‘Area’ feature.

**Fig 13:**
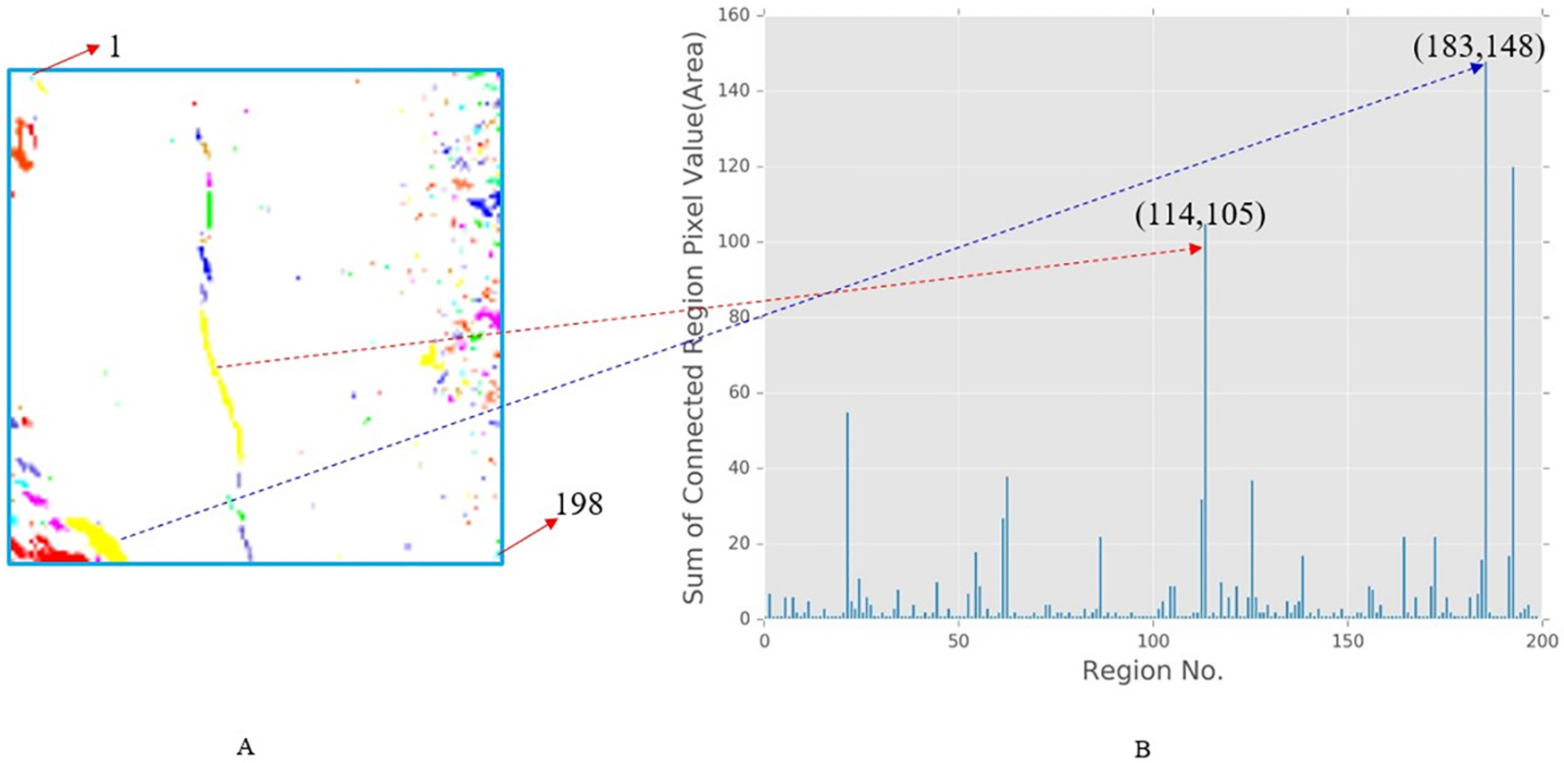
The correlation of different regions with ‘Area’ feature. (A) An image with coloured labelling. (B) The region and its ‘Area’ value.

As shown in the Fig 12B, the 8-connected components (or called 8-neighborhood) are defined as regions of adjacent pixels that have the same input value (in this paper, the value is 0 or 1). According to the definition of 8-neighborhood, we labelled the every connected regions. And the different colours in the Fig 12C represent different independent regions. Therefore, in order to extract features to distinguish between coal and rock surface cracks and other non-crack objects, the segmentation of the images needs to be labelled in left-to-right and top-to-bottom order according to the definition of the 8-connected components. As shown in the Fig 13A, the arrow in the upper left indicates the 1st region, and the arrow in the lower right represents the last region (in this figure is 198th region). In the label processing, there is an implied order from the upper left to the lower right.

Furthermore, in the Fig 13B we can see that the area value of 114th region and 183th region both satisfy the condition about ‘Area’ feature (the area value of 114th region is 105 and the area value of 183th is 148), however, the shape of them is entirely different, one belongs to the crack, and the other is non-crack.

From the Figs 12 and 13, we can observe that the prerequisite of crack formation is that the area of the region must meet certain values. This feature can further remove many unrelated regions as shown in Fig 12D. However, in the regions marked above, when the feature of the area is satisfied, it may be caused by the detachment of the surface of the coal and rock mass itself or other factors rather than experimentation. Therefore, further extraction of the feature is required, and this paper propose 8 features based on 3 aspects according the calculation formula: pixel aspect (area and maximum diameter are calculated by the region pixel value), statistics aspect (distance mean, distance deviation and roundness calculated by the mean and variance of a region) and morphological aspect (anisometry, bulkiness and compactness are calculated by the region shape). We use these 8 parameters as features to judge a region is a crack or not as the shape of crack is complicated and it's hard to fully represent a crack using a single feature. On the other hand, we know that using the more features will not sure get better result. As some features are redundant and different features has different weights. We will discuss it in the next analysis of crack feature parameter section.

We use *p* denotes pixel value of the centre of a certain region, *p*_*i*,*j*_ denotes the pixel value of a pixel which coordinate is (*i*, *j*) in the region.

**Area**: The area is defined as the number of pixels of a region, which is denoted by S. This is the most important feature parameter to judge the crack, but there are two problems in the selection of the area value. First, it is difficult to accurately determine a threshold because if the threshold is too large, it will allow too many non-cracks to occur; if the threshold is too small, it will not select all cracks. Second, even if the area conditions are met, there are some regions are not belong to crack, e.g. the 183th region in the Fig 13.

**Maximum diameter**: This feature parameter is the maximum diameter of the region. This feature parameter is the maximum diameter of the contour. In the case where the area parameters meet, the maximum diameter is a more intuitive parameter. The calculation procedure is to compute the distance between the two pixels of a region’s border in a circular manner. And the max value is the maximum diameter of a region.

**Dist_mean**: Mean distance from the region border to the centre.

$$dist_{mean}=\frac{1}{S}\sum \left|\left|p-p_{i}\right|\right|$$ (2)

**Dist_deviation**: Deviation of the distance from the region border to the centre.

$$dist_{deviation}=\;\sqrt{\frac{1}{S}\sum {\left(\left|\left|p-p_{i}\right|\right|-{\text{dist}}_{\text{mean}}\right)}^{2}}$$ (3)

**Roundness**: The operator roundness examines the distance between the contour and the centre of the area. In particular, the mean distance (dist_deviation), the deviation from the mean distance (dist_mean) and the two shape features that are derived are determined. Roundness is the relationship between mean value and standard deviation.

$$Roundness=1-\;\frac{dist_{mean}}{dist_{\text{deviation}}}$$ (4)

In addition to extract features in the pixel level and statistic level, we also use the shape of the crack itself as features. First, we calculate the geometric moments *M*_11_, *M*_20_ and *M*_02_ of the every regions. In this work, *r*_0_ and *c*_0_ are the coordinates of the centre of gravity of a region *R*. Then the moments *M*_*i*,*j*_ are defined by:

$$M_{i,j}=\Sigma_{\left(r,c\right)\in R}{\left(r_{0}-r\right)}^{i}{\left(c_{0}-c\right)}^{j}$$ (5)

The radii *R*_*a*_ and *R*_*b*_ are calculated as:

$$R_{a}=\;\frac{\sqrt{8\left(M_{20}+M_{02}+\sqrt{{\left(M_{20}-M_{02}\right)}^{2}+4M_{11}^{2}}\right)}}{2}$$ (6)

$$R_{b}=\;\frac{\sqrt{8\left(M_{20}+M_{02}-\sqrt{{\left(M_{20}-M_{02}\right)}^{2}+4M_{11}^{2}}\right)}}{2}$$ (7)

**Anisometry**: Anisometry of the contours or polygons.

$$Anisometry\;=\;\frac{R_{a}}{R_{b}}$$ (8)

**Bulkiness**: Bulkiness of the contours or polygons

$$Bulkiness\;=\;\frac{\pi \cdot R_{a}\cdot R_{b}}{S}$$ (9)

**Compactness**: The operator compactness calculates the compactness of the input regions.

$$C^{\prime }\;=\;\frac{L^{2}}{4\cdot S\cdot \pi }\;,\;compactness\;=\;\text{max}\left(1,C^{\prime }\right)$$ (10)

where L is the length of the contour, and the shape factor C of a circle is 1.

The extracted region feature is stored in a two-dimensional array where each row represents the sequence number of each region each column represents the eight features of each region. Then, 300 crack regions and 300 non-crack regions are extracted as the training data of SVM according to the research and definition of surface crack of coal and rock mass.

### 3.4. SVM

The fundamental idea of SVM (Support Vector Machine) is to construct a hyper-plane as the decision line that separates the classes with the largest margin (introduced by Cortes C et al. (1998) [22] and Vapnik V et al. (1998) [23]).

In classification, suppose there are m (m = 480) samples in the training data corresponding to two groups, and each sample is denoted by a vector ***x***_***i***_ (*i* = 1, …, m)and each vector has 8 items that represent the selected crack features. In addition, a vector **y** ∈ (−1, 1) denotes the two classes of crack and non-crack region.

The input vector Si is mapped into a high dimensional feature space H by using suitable kernel function k(x_i, x_j) for non-linear training data. The popular kernels, Gaussian radial basis function (rbf) and linear, used in this study are defined mathematically by:

$$k\left(x_{i},\;\;x_{j}\right)=\text{exp}\left(-\frac{{\left\|x_{i}-x_{j}\right\|}^{2}}{2\sigma^{2}}\right),\;\;k\left(x_{i},\;\;x_{j}\right)=\;x_{i}^{T}x_{j}$$ (11)

The classification function, f(x), is determined using training data and then will be used to classify the unseen test data set. This function is defined in terms of kernels:

$$f\left(x\right)=sgn\left[\sum \alpha_{i}y_{i}\text{k}\left(x_{i},\;x_{j}\right)+b\right]$$ (12)

where b is a bias term, and *α*_*i*_ is the Lagrange multiplier coefficient obtained by solving the quadratic programming problem. Mathematically (introduced by Lkopf et al. (1999) [24]).

$$\text{Maximiz}:\text{W}\left(\alpha \right)\;=\Sigma_{i=1}^{m}\;\alpha_{i}-\;\frac{1}{2}\Sigma_{i=1}^{m}\Sigma_{j=1}^{m}y_{i}y_{j}\text{k}\left(x_{i},\;x_{j}\right)\alpha_{i}\alpha_{j}$$ (13)

under the constraints of: $0\;\leq \;\alpha_{i}\;\leq C,\;\left(i=1,\;\ldots ,\;m\right)\;and\;\Sigma_{i=1}^{m}y_{i}\alpha_{i}=0$, where C is a non-negative regularization parameter.

## 4. Results and discussion

### 4.1 Crack detection accuracy

After both the image dataset and the model are ready, the SVM classifier can be imported for training and testing. The results of the final tests are shown in Table 2.

**Table 2:** Accuracy of the image dataset.

Input Data	SVM Kernel	
	rbf	linear
***D***_***image***_	57.1%	56.3%
***D***_***image***_ **with Segmentation**	63.8%	68.7%

As shown in Table 2, if the image is directly input into the classifier, it will not get a good effect. Therefore, in this paper, the images in ***D***_***image***_ are segmented and extracted; then, 300 regions representing the cracks and 300 regions representing non-cracks are labelled ‘1’ and ‘-1’. After the random distribution in accordance with the ratio of 8:2, we obtain the training set, which has 480 regions, and the test set, which has 120 regions. Finally, the new data set is input into the SVM classifier for training and testing. The results of the final tests are shown in Table 3.

**Table 3:** Accuracy of the region dataset.

Input Data	SVM Kernel	
	rbf	linear
***D***_***region_train***_	98.5%	97.3%
***D***_***region_test***_	95.0%	94.1%

As shown in Table 3, after extracting the crack feature, the accuracy of the classifier is nearly 95%. To further analyse the results of the experiment, we calculate the confusion matrix (shown in Table 4) of the SVM classifier result. Each column of the confusion matrix represents an instance prediction of a class, and each row represents an instance of an actual class. In artificial intelligence, the confusion matrix is a visualization tool, especially for supervised learning.

**Table 4:** Confusion matrix.

Actual	Predicted	
	Crack (1)	Non-crack (-1)
**Crack (1)**	True Positive (TP)	False Negative (FN)
**Non-crack (-1)**	False Positive (FP)	True Negative (TN)

As shown in Table 5, four samples representing non-crack are classified as crack, and three samples representing crack are classified as non-crack in the type of ‘linear’. In addition, in the type of ‘rbf’ kernel, only one sample representing non-crack is classified as crack, and five samples representing crack are classified as non-crack. To compare the results of the two classifiers models more clearly, the results of the confusion matrix are used to calculate the precision and recall of the models.

**Table 5:** Confusion matrix of *D*_*region_test*_.

Input data	Confusion Matrix
***D***_***region_test***_ **with ‘linear’**	$\left(\begin{array}{cc} 58 & 3\\ 4 & 55 \end{array}\right)$
***D***_***region_test***_ **with ‘rbf’**	$\left(\begin{array}{cc} 49 & 5\\ 1 & 65 \end{array}\right)$

Positive predictive value (Precision):

$$P=\;\frac{\text{TP}}{\text{TP}+\text{FP}}$$ (14)

True positive rate (Recall):

$$R=\;\frac{\text{TP}}{\text{TP}+\text{FN}}$$ (15)

In general, the precision and recall rate is a contradictory variable, when the precision rate is higher; the recall rate is often low; vice versa. The results are shown in the Table 6.

**Table 6:** Precision and recall value of *D*_*region_test*_.

	P	R
***D***_***region_test***_ **with ‘Linear’**	93.5%	95%
***D***_***region_test***_ **with ‘rbf’**	98%	90.7%

**Fig 14:**
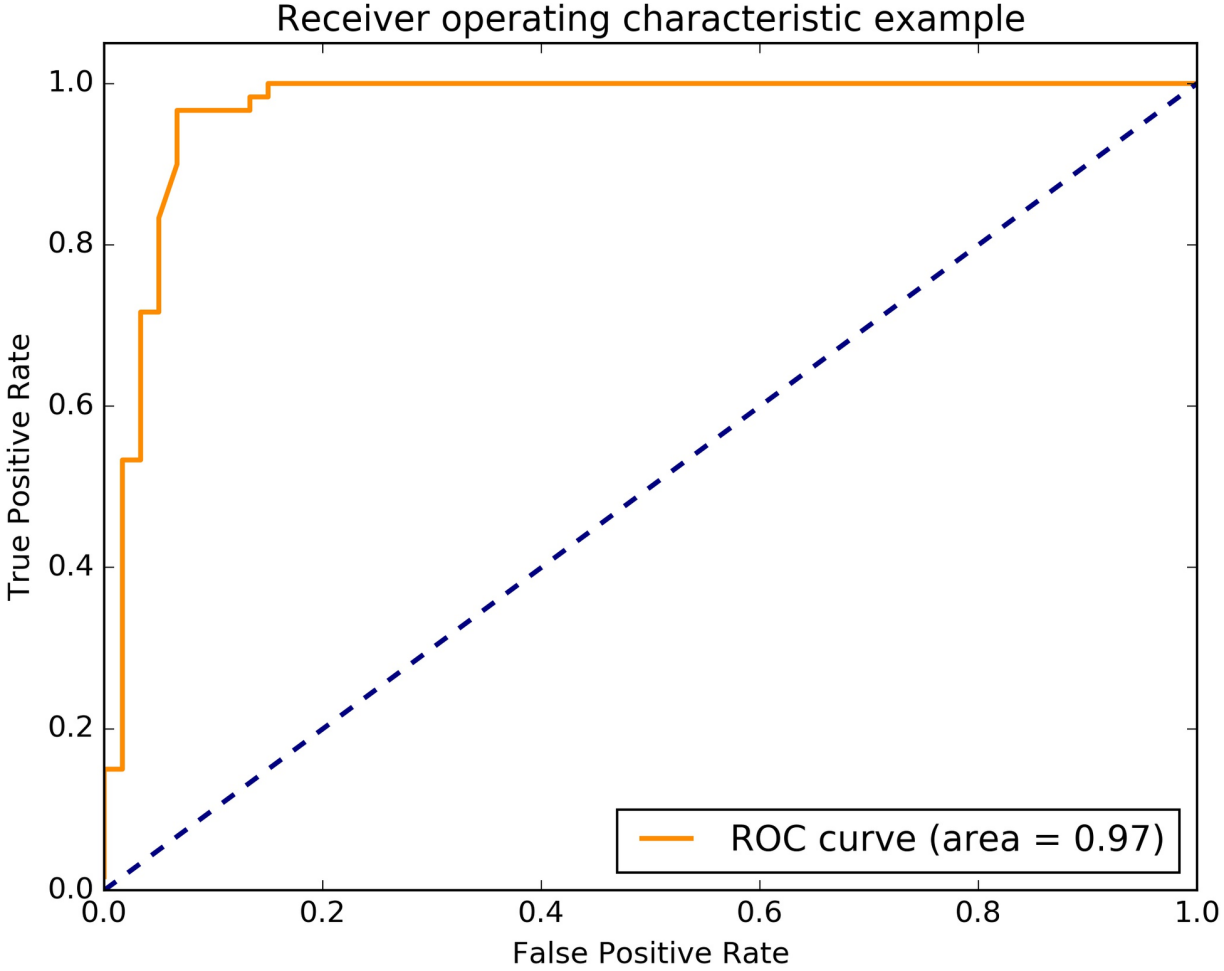
The ROC of linear kernel classifier.

In the mining process, it is more desirable to minimize the leakage of cracks; therefore, the recall rate is more important, and we choose the linear kernel. A receiver operating characteristic (ROC) is a graphical plot that illustrates the performance of a binary classifier system as its discrimination threshold is varied. The ROC curve of the training classifier is shown in Fig 14.

**Fig 15:**
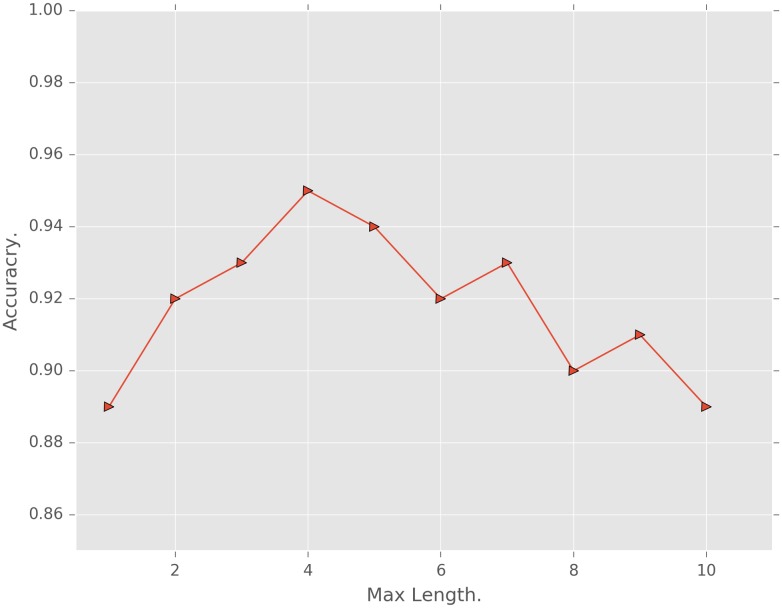
Crack detection accuracy with different maximum lengths.

In the experiment, we also found that image segmentation is a key step that directly affects the crack detection accuracy. In the hysteresis threshold algorithm used in this paper, the effect of the maximum length on the accuracy is shown in Fig 15. We can observe that when the maximum length is set at 4, the crack detection accuracy is the highest.

### 4.2 Analysis of crack feature parameter

**Fig 16:**
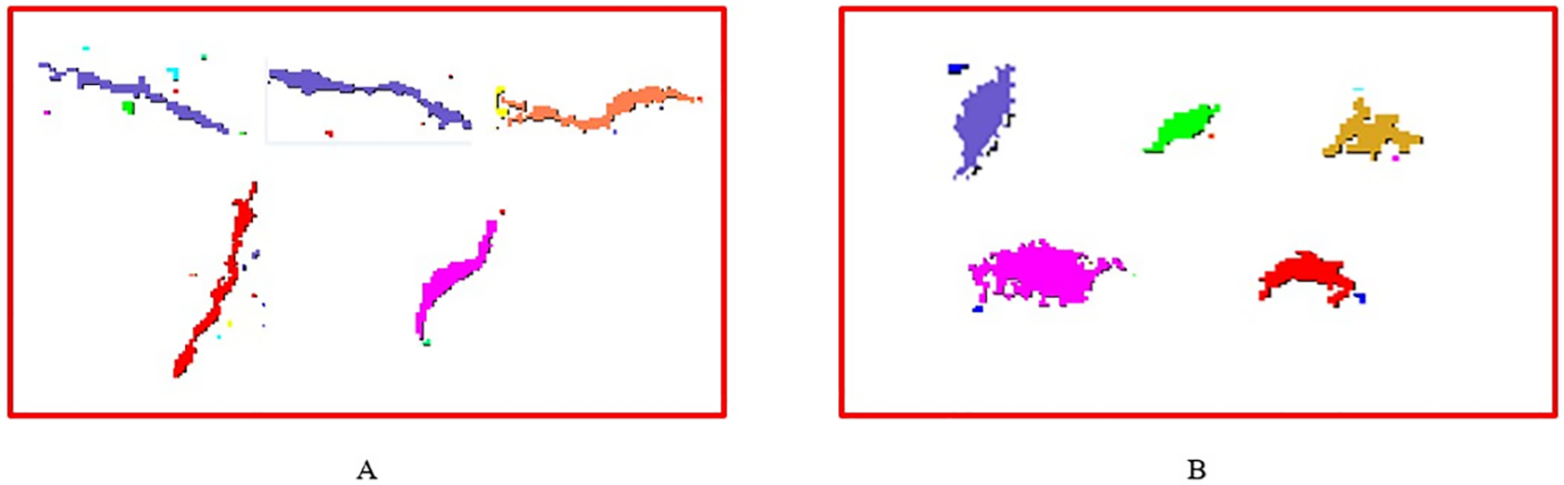
Examples of crack and non-crack. (A) Examples of a crack shape; (B) Examples of a non-crack shape.

To accurately describe the effect of the crack feature parameters on the accuracy of crack detection, five sets of crack and five sets of non-crack parameters are shown in Tables 7 and 8, and their shapes are shown in Fig 16.

**Table 7:** Crack feature parameter.

No.	Area	Max diameter	Dist deviation	Dist mean	Roundness	Anisometry	Bulkiness	Compactness
1	74.00	36.68	5.03	8.25	0.39	8.54	1.98	9.05
2	155.0	27.78	3.81	7.38	0.48	2.49	1.57	4.13
3	226.0	54.00	7.45	14.12	0.47	4.76	2.51	9.76
4	197.0	68.95	9.91	16.26	0.39	11.44	2.15	11.73
5	183.0	44.29	5.9	11.39	0.48	4.98	1.60	4.71

**Table 8:** Non-crack feature parameter.

No.	Area	Max diameter	Dist deviation	Dist mean	Roundness	Anisometry	Bulkiness	Compactness
1	107.0	21.54	2.40	5.73	0.58	2.88	1.05	1.80
2	77.0	18.43	2.03	4.64	0.56	2.78	1.09	2.05
3	104.0	16.49	1.78	4.73	0.62	1.24	1.41	3.41
4	509.0	44.01	4.07	12.81	0.68	1.94	1.18	5.56
5	133.0	18.60	2.34	6.25	0.62	1.38	1.59	3.52

Intuitively, in the case of an area parameter greater than a certain value, a crack should have a longer diameter and a smaller roundness value than a non-crack. However, as shown in Fig 16, the maximum diameter and roundness of crack and non-crack are similar and cannot be distinguished well. Therefore, when the area and max diameter parameters cannot distinguish whether the region is crack or not, we need to find other parameters to judge, and it appears that the dist_deviation, anisometry and compactness parameters of the crack and non-crack have the largest differences in Tables 7 and 8 and can be more useful in the classification.

**Fig 17:**
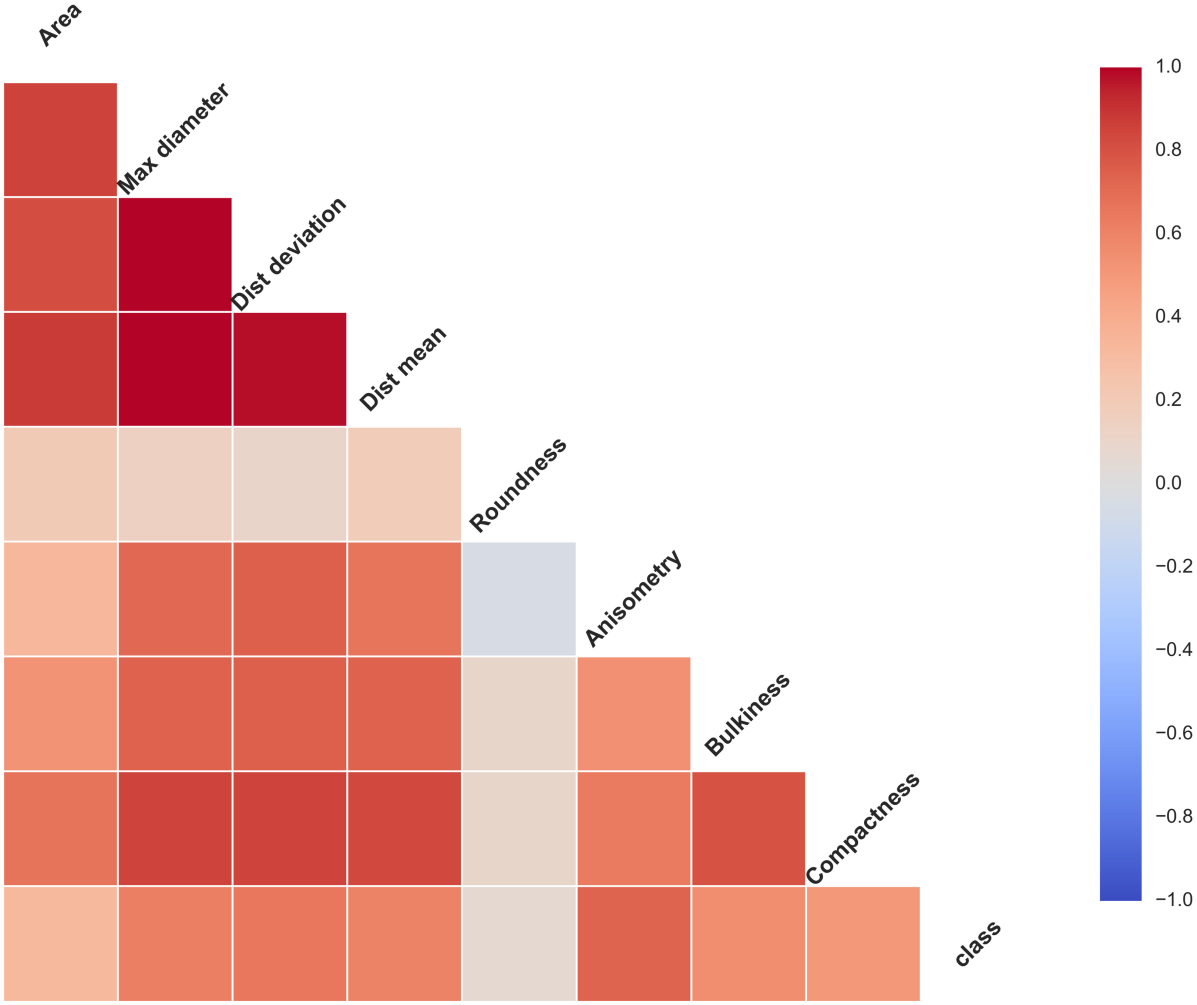
The correlation matrix of features and class.

In order to analyse the correlation of different features and which feature has greatest influence on the result, we use Correlation Feature Selection (CFS) method and plot correlation matrix using a python library (seaborn 0.7.1) as shown in the Fig 17. The CFS measure evaluates subsets of features on the basis of the following hypothesis: Good feature subsets contain features highly correlated with the classification, yet uncorrelated to each other [25–26].

As we can see in the Fig 17, the grids in the bottom row represent the relationship between different features and the class (crack or non-crack). And different grids represents different features. With the correlation increase, the colour of the grid gets deeper.

We can observe from Fig 17, feature roundness has minimal effect on the result and feature anisometry is strongly correlated with the class (it is informative). Furthermore, these three features (maximum diameter, dist_deviation and dist_mean) are strongly correlated. We have thus some redundant features. In subsequent work, we can reduce the extraction of the redundant feature to reduce the recognition time.

### 4.3 Classifier accuracy test

**Fig 18:**
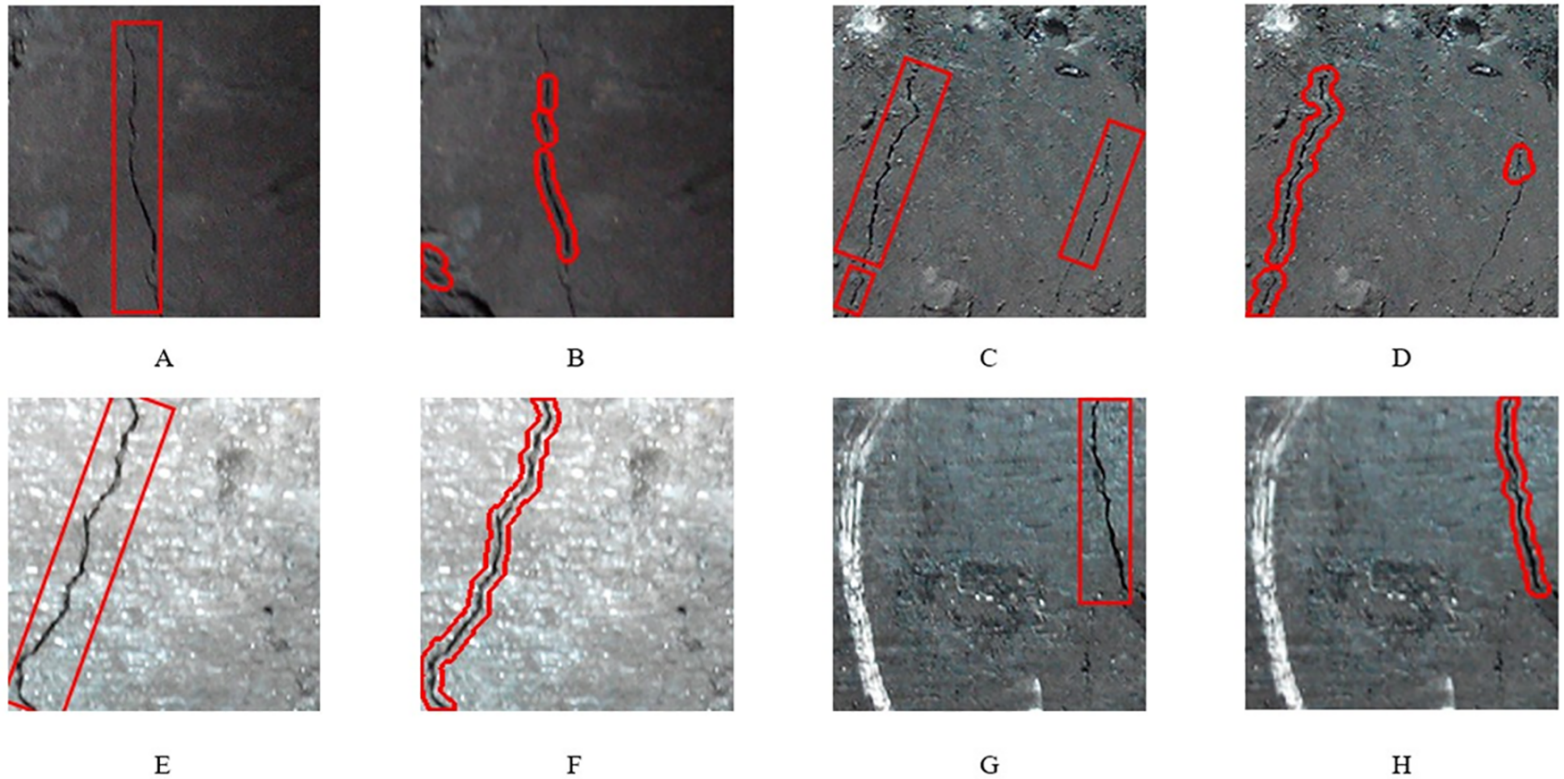
Examples of practical detection results. (A), (C), (E) and (G) are original images with visual detection results; (B), (D), (F) and (H) are automatic detected results.

According to the above crack detection algorithm, each image in *D*_*image*_ is segmented and classified. When a region is detected as belonging to a crack, a morphological dilatation operation was used for the identified crack. Fig 18 gives four examples of the practical application of the SVM classifier.

In general, over 90% crack lengths are correctly detected, such as Fig 18F and 18H. However, there are still some misidentified objects. In Fig 18B, a non-cracked region is incorrectly identified as a crack in the lower left corner of the figure. In addition, in Fig 18D, on the right side of the figure, there is a long crack in the original image, but only a small part is identified. The main reason for the detection error in Fig 18B and the missing detection in Fig 18D is that in the process of segmentation, a long crack is divided into many small parts; therefore, the classifier cannot work properly.

## 5 Conclusions

A method for detecting the surface cracks for loaded coal using a vibration failure process based on a vibration failure test system and SVM was proposed and developed. According to the characteristics of the surface cracks on coal and rock mass, histogram equalization and a hysteresis threshold algorithm were used to reduce the noise and emphasize the crack. Then, a detailed description of the crack feature extract and model training steps are given in the above sections. This led to a significant improvement in the classification accuracy. The test results show that the proposed algorithm and model can effectively detect surface cracks on coal and rock mass. The proposed method is easy to carry out and effective, and the proposed eight features of surface cracks may be suitable for other pattern recognition.

During the experiment, we also found some shortcomings of the algorithm; thus, some work needs to be continued. First, using principal component analysis (PCA) to further analyse which features of the crack play a major role will be useful so that we can greatly reduce the run-time of the program. Second, this paper only identifies whether a region is cracked or non-cracked; thus, in the next step, we can judge the type of crack, which may be more helpful in predicting the risk of coal and rock dynamic disasters.
